# Insight into the Inhibitory Mechanism of Embryonic Ectoderm Development Subunit by Triazolopyrimidine Derivatives as Inhibitors through Molecular Dynamics Simulation

**DOI:** 10.3390/molecules28247997

**Published:** 2023-12-07

**Authors:** Jianan Ju, Hao Zhang, Shanshan Guan, Chang Liu, Juan Du, Xiaoli Shen, Song Wang

**Affiliations:** 1 Institute of Theoretical Chemistry, College of Chemistry, Jilin University, 2 Liutiao Road, Changchun 130023, China; jujn21@mails.jlu.edu.cn (J.J.); stringbell@jlu.edu.cn (H.Z.); changliu20@mails.jlu.edu.cn (C.L.); dujuan19@mails.jlu.edu.cn (J.D.); shenxl22@mails.jlu.edu.cn (X.S.); 2 College of Biology and Food Engineering, Jilin Engineering Normal University, Changchun 130052, China; guanshanshan@jlenu.edu.cn; 3 Key Laboratory of Molecular Nutrition at Universities of Jilin Province, Changchun 130052, China

**Keywords:** EED, triazolopyrimidine derivatives, molecular dynamics simulation, MM/PBSA, inhibitory mechanism

## Abstract

Inhibition of the Embryonic Ectoderm Development (EED) subunit in **Polycomb Repressive Complex 2** (PRC2) can inhibit tumor growth. In this paper, we selected six experimentally designed EED competitive Inhibitors of the triazolopyrimidine derivatives class. We investigated the difference in the binding mode of the natural substrate to the Inhibitors and the effects of differences in the parent nuclei, heads, and tails of the Inhibitors on the inhibitory capacity. The results showed that the binding free energy of this class of Inhibitors was close to or lower compared to the natural substrate, providing an energetic basis for competitive inhibition. For the Inhibitors, the presence of a strong negatively charged group at the 6-position of the parent nucleus or the 8′-position of the head would make the hydrogen atom on the head imino group prone to flip, resulting in the vertical movement of the parent nucleus, which significantly decreased the inhibitory ability. When the 6-position of the parent nucleus was a nonpolar group, the parent nucleus would move horizontally, slightly decreasing the inhibitory ability. When the 8′-position of the head was methylene, it formed an intramolecular hydrophobic interaction with the benzene ring on the tail, resulting in a significant increase in inhibition ability.

## 1. Introduction

**Polycomb Repressive Complex 2** (PRC2) is a conserved multi-protein, repressive chromatin complex essential for developing and maintaining eukaryotic organisms. PRC2 mediates mono-methylation, di-methylation, and tri-methylation of lysine 27 on histone H3 (H3K27me1/2/3), of which H3K27me3 is a repressor mark associated with chromatin compression and site-specific gene silencing [1,2,3,4]. As a critical epigenetic modulator, PRC2 plays a role in multiple biological processes [5], such as cell proliferation, stem cell self-renewal, differentiation, DNA repair, and epithelial-to-mesenchymal transition [6]. However, PRC2 overexpression and activating mutations in PRC2 individual subunits can cause various cancers, including prostate cancer [7], lung cancer [8,9], breast cancer [10], liver cancer [11], gastric cancer [12], ovarian cancer [13], and so on [14]. Therefore, the inhibition of PRC2 activity can inhibit the growth of certain cancers, making it an attractive target for cancer therapy [15,16].

**Figure 1:**
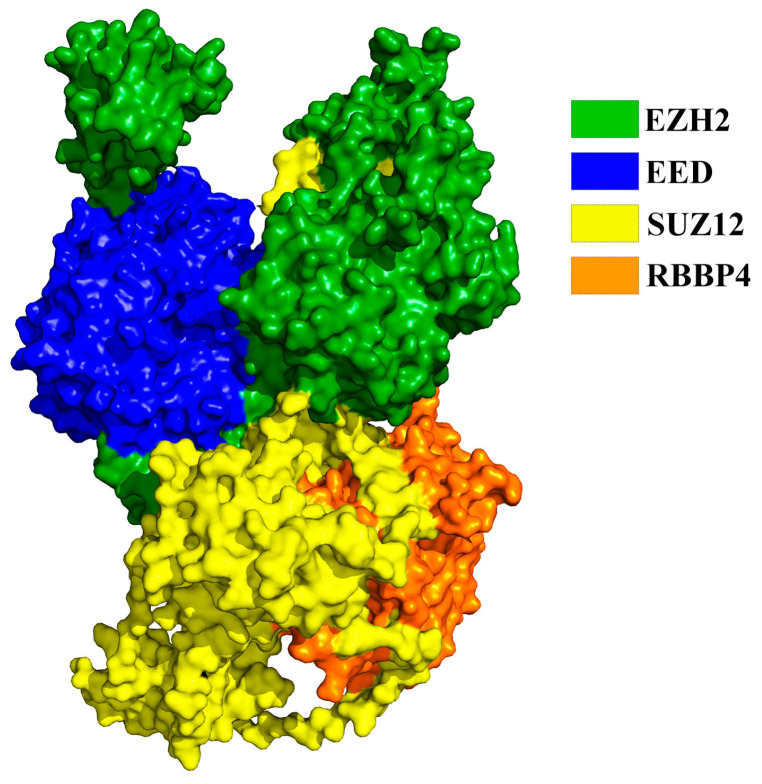
Conformation of the core subunit in PRC2 protein (PDBID:6c24).

The core subunits of PRC2 are composed of Enhancer of Zeste Homolog 2 (EZH2) or its highly related homolog EZH1, Embryonic Ectoderm Development (EED), Suppressor of Zeste 12 (SUZ12), and Retinoblastoma Binding Protein4/7 (RBBP4/7), shown in Figure 1 [17]. One of them, EZH1/2, has histone methyltransferase activity and is responsible for transferring methyl from the natural methyl donor cofactor S-adenosylmethionine (SAM) to the ε-amino group of the acceptor H3K27. However, it must be catalytically active in the presence of EED and SUZ12. Once EZH1/2 trimethylates H3K27 on a specific nucleosome, the resulting H3K27me3 product binds to EED. This binding then triggers EZH1/2 to denature, making the next catalytic step easier. This process is called the denatured activation of PRC2 [18]. Multiple groups have developed inhibitors targeting EZH2, the primary catalytic subunit in PRC2. Some of these Inhibitors are dual Inhibitors that target both EZH1 and EZH2. This class of Inhibitors competes with SAM and has demonstrated antitumor activity in preclinical and clinical models [19,20,21]. However, new research indicates that when treated with SAM-competitive EZH2 inhibitors, different lymphoma cancer cell lines can develop new mutations in both the wild-type and mutant EZH2. These mutations disrupt the binding of EZH2 inhibitors to PRC2, resulting in PRC2-acquired resistance to these catalytic inhibitors [22,23,24]. These occurrences make current inhibitors that target EZH2 inadequate to inhibit its cancer-causing activity. Based on the catalytic process, the binding of EED to H3K27me3 is crucial for PRC2 [25]. Therefore, targeting the EED subunit could be a promising new approach to inhibiting PRC2 and may offer a solution to the challenges associated with EZH2 inhibitors [26].

**Figure 2:**
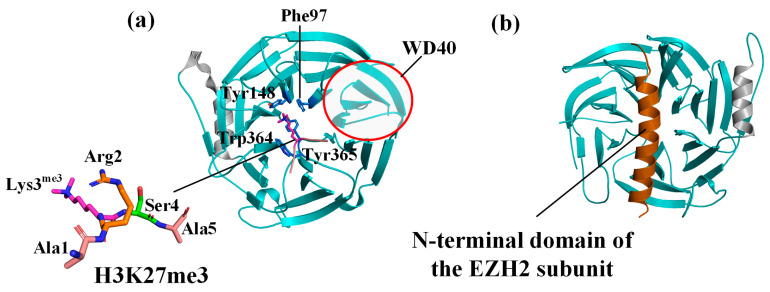
Conformations of the EED protein. (**a**) The EED top surface contains H3K27me3 (PDBID:3IIW), where the four light blue residues represent aromatic cage residues and the pink portion is the natural substrate (**b**) and the EED bottom surface contains the N-terminal structural domain of the EZH2 subunit (PDBID:7QK4).

EED is a protein containing the WD40 repeat domain, and its C-terminal (81–441 residues) has seven WD40 repeat groups. This motif consists of approximately 40 residues and forms a four-stranded antiparallel β-folded sheet [27]. The repeat seven WD40 domains fold into a typical seven-bladed β-propeller structure. Each surface of the EED has a central pocket with a smaller plane on the top surface and a larger plane on the bottom surface. The co-crystal structure of EED bound to a trimethylated pentapeptide (Ala1-Arg2-Lys3^me3^-Ser4-Ala5) truncated from H3K27me3 is shown in Figure 2a [25]. This Complex shows that EED has an “aromatic cage” consisting of four aromatic amino acids (Phe97, Tyr148, Trp364, and Tyr365) on its top surface. The ARL^me3^SA side chain is inserted into this “aromatic cage” and remains to stabilize the combination [28]. Furthermore, the bottom surface of EED can bind to the N-terminal EBD structural domain (residues 39–68) of EZH2 in Figure 2b [29,30].

**Figure 3:**
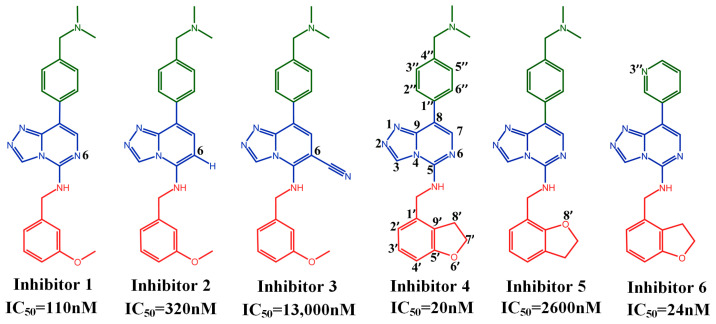
The structures of the six Inhibitors include the ring’s atom numbering and corresponding IC_50_ value.

After the trimethylation of lysine 27 on histone H3 by EZH2, by binding its H3K27me3 product via EED, PRC2 can spread to neighboring nucleosomes to deposit H3K27me3, initiating a positive feedback loop mechanism that efficiently propagates the repressive H3K27me3 histone mark on chromatin. H3K27me3, as a gene silencing marker, can cause various cancers, so inhibiting the interaction between EED and H3K27me3 can effectively inhibit tumor production [5]. Andreas Lingel et al. had proven that compounds that occupied the central pocket on the top surface of EED, which bounds to H3K27me3, could inhibit the EED-H3K27me3 interaction [31]. At present, inhibitors targeting EED mainly include EED226, EEDi-5285, EEDi-5273, and MAK683. EED226 achieved complete tumor regression at a dose of 300 mg/kg in the KARPAS300 xenograft model [32]. In addition, the literature has shown that EED226 could inhibit excessive histone trivalent methylation in liver cancer cells, thereby relieving the inhibition of the mediator of cell death (Bim) and cyclin-dependent kinase inhibitor 1A (p21)’s expression and achieving the effect of inhibiting liver cancer cell proliferation [33]. The Inhibitor EEDi-5285 exhibited excellent pharmacokinetics (PK) characteristics in mice, achieving complete and persistent tumor regression in the KARPAS422 xenograft model at an oral dose of 50 mg/kg [34]. EEDi-5285 was 100 times more potent in binding to EED than the Inhibitor EED226 and 300 times more potent in inhibiting cell growth than the Inhibitor EED226. This suggested that inhibitors with a strong ability to bind to EED had a greater capacity for growth regulation at the cellular level. The Inhibitor EEDi-5273 exhibited good PK and absorption, metabolism, distribution, and excretion (ADME) characteristics, and could achieve complete and persistent tumor regression at 50 mg/kg in the KARPAS422 xenograft model without any signs of toxicity [35]. Huang et al. progressively optimized the Inhibitor EED226 to obtain a more potent and less toxic Inhibitor, MAK683, which was able to achieve complete tumor regression in nude mice at 10 mg/kg in the Karpas 422 xenograft model and was well tolerated at relevant plasma exposure levels [30]. Their optimization process also resulted in the synthesis of a series of triazolopyrimidine derivatives with the same parent nucleus as EED226, and these compounds also possessed competitive inhibitory abilities of the EED protein. However, the mechanism of inhibition of this class of inhibitors at the molecular level was not known. Therefore, in the present work, we chose six of these representative derivatives as subjects for the study of this class of inhibitors. The six Inhibitors have the structure shown in Figure 3 (the atomic numbers of each part are also labeled in the figure), in which the red part is called the head, the blue part is called the parent nucleus, and the green part is called the tail. The different substitutions of the three parts could result in a more than 100-fold differences in inhibition ability between the Inhibitors. Furthermore, Huang et al. utilized a molecular docking method to examine the interaction between the EED protein and Inhibitors 4 and 5 on a molecular scale [30].

Huang et al. also utilized a molecular docking method to examine the interaction between the EED protein and Inhibitors 4 and 5 on a molecular scale [30]. However, the protein was rigid in the docking calculations and could only be studied qualitatively, which did not give binding energies. Additionally, this approach did not clarify why various inhibitors had different abilities to inhibit the EED protein. To address the above problems, six representative Inhibitors of the triazolopyrimidine derivatives, as shown in Figure 3, divided into three comparison groups; among them, Inhibitor 1, Inhibitor 2, and Inhibitor 3 differed only in the substituent of the 6-position on the parent nucleus as the first group; Inhibitor 4 and Inhibitor 5 differed only in the substitution position of the oxygen atom in the head as the second group; and Inhibitor 4 and Inhibitor 6 differed only in the tail as the third group. Using molecular dynamics simulations and binding free energy calculations, we could examine the binding modes and energies of PRC2 with six Inhibitors. Additionally, by analyzing the Inhibitors within each group, we could explore how different substituent groups in the head, parent nucleus, and tail affected their inhibitory ability. The findings of this study can provide valuable guidance for creating new inhibitors.

## 2. Results

### 2.1. Molecular Docking Results

**Figure 4:**
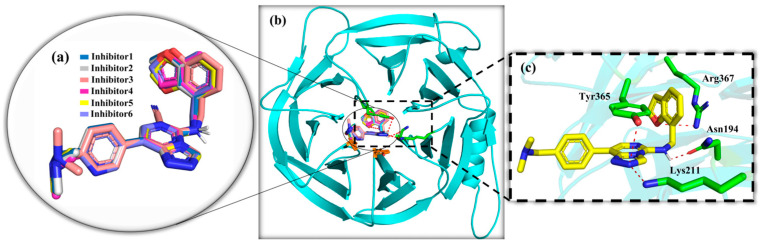
The binding position of inhibitors and the formation of hydrogen bonds in EED. (**a**,**b**) The binding positions of the Inhibitors in the EED, and the circular magnification frame detail the comparison of the poses of the six Inhibitors, where the atoms marked in dark red are oxygen atoms. (**c**) The square magnification frame shows the hydrogen bonding formed between the Inhibitors and the surrounding residues after docking (in the case of inhibitor 2, which had the highest number of hydrogen bonds), where the red dotted lines represent hydrogen bonds and the portion labelled green is the residues that form hydrogen bonds with Inhibitor 2.

Molecular docking results showed that the six Inhibitors were all docked at specific binding sites in aromatic cages of the EED protein and had similar postures (Figure 4a,b). The affinity of Inhibitors 1–6 was −10.2, −10.4, −9.8, −10.7, −10.4, −11.0 kJ·mol^−1^. All the Inhibitors formed hydrogen bonds to Asn194, Lys211, and Tyr365 in the protein, and Inhibitor 2 also included additional hydrogen bonds to Arg367 (Figure 4c). This result is generally accorded with previous docking results in the literature, differing only in the absence of any mention of the hydrogen bond formed by Arg367 with Inhibitor 2 [30].

### 2.2. Stability Analysis of the Simulation System

**Figure 5:**
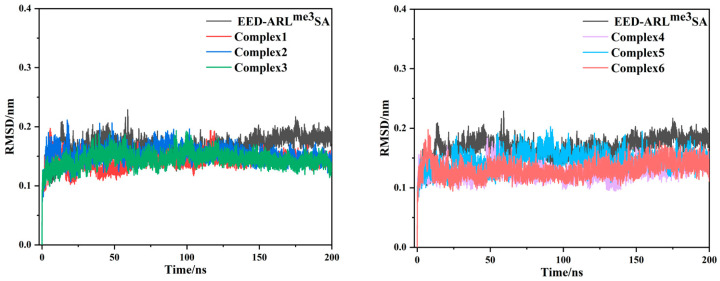
RMSD diagram of the C_α_ atomic skeleton of six complex systems and EED-ARL^me3^SA with simulated time.

After 200 ns of molecular dynamics simulations for each of the six EED-inhibitor complexes as well as for the EED-pentapeptide complex (EED-ARL^me3^SA), we have selected one set of parallel simulations from two sets of each system to be discussed (the results obtained from copies were not identical to the master samples, but the main conclusions were basically the same, so we did not discuss them in detail). The Root Mean Square Deviation (RMSD) during the simulation of the seven systems with the C_α_ atom in the initial conformation as a reference is shown in Figure 5. As can be seen from the figure, the RMSD of each system tends to stabilize after an initial rapid increase and reaches equilibrium after 100 ns, and the fluctuation of the RMSD after equilibrium is less than 0.1 nm.

### 2.3. Free Energy Landscape and Sampling

**Figure 6:**
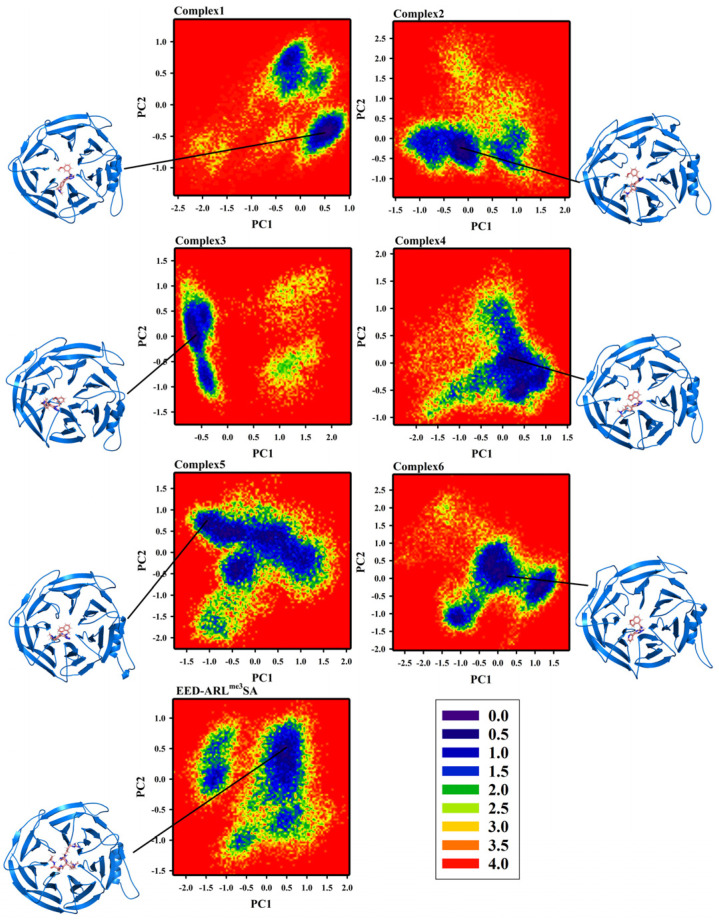
Free energy landscape and sampling of seven complex systems.

The simulation trajectories were projected onto the first two eigenvectors, PC1 and PC2, to create a free energy landscape (FEL), which reflected the changes in conformational energies in the simulated trajectories. The FEL of the six complex systems and EED-ARL^me3^SA are shown in Figure 6, where the darker blue color represents the lower energy of the conformation. We sampled from the clusters with the lowest free energy and region where the RMSD was as smooth as possible, as the primary goal of the later conformational analysis. Based on the two conditions, seven samples of the simulation systems for the corresponding simulation time were 152.73 ns, 157.30 ns, 116.99 ns, 101.53 ns, 111.16 ns, 136.20 ns, and 62.06 ns. The 3D conformations of the seven samples can be found in Supplementary Materials’ complexes 1~6.pdb and EED-ARL^me3^SA.pdb. The 3D conformations of all copies are also placed in Supplementary Materials.

### 2.4. Sample Conformation Analysis

**Figure 7:**
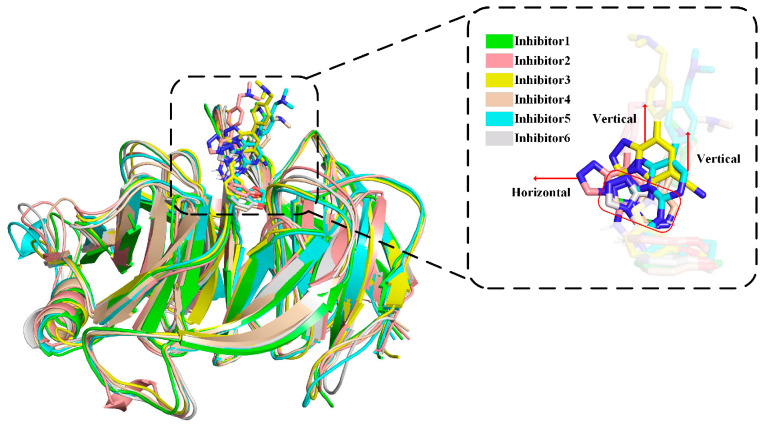
Complexes overlap the diagram of six samples, where the atoms marked in dark red are oxygen atoms. The square magnification frame is the overlap of six Inhibitors, and the red rectangular frame shows the location of the parent nucleus in Inhibitors 1, 4, and 6.

Comparing the sample conformation with the initial conformation obtained by docking, in **Complexes 1**, **4**, and **6**, the binding positions of Inhibitors differed little from the initial conformation for Inhibitors. In **Complexes 3** and **5**, the Inhibitors moved out of the binding pocket vertically, as shown in the enlarged box in Figure 7, making the part inserted into the binding pocket shorter, especially since the vertical movement of Inhibitor 3 was more pronounced than Inhibitor 5. This vertical movement caused the parent nucleus of the Inhibitor to separate from the aromatic cage to some extent and made the tail trimethylamine group more into the solvent. In **Complex 2**, the Inhibitor underwent horizontal movement perpendicular to the direction of motion in **Complexes 3** and **5** (Figure 7). Unlike the vertical movement, this horizontal movement did not separate the parent nucleus of the Inhibitor from the aromatic cage.

**Figure 8:**
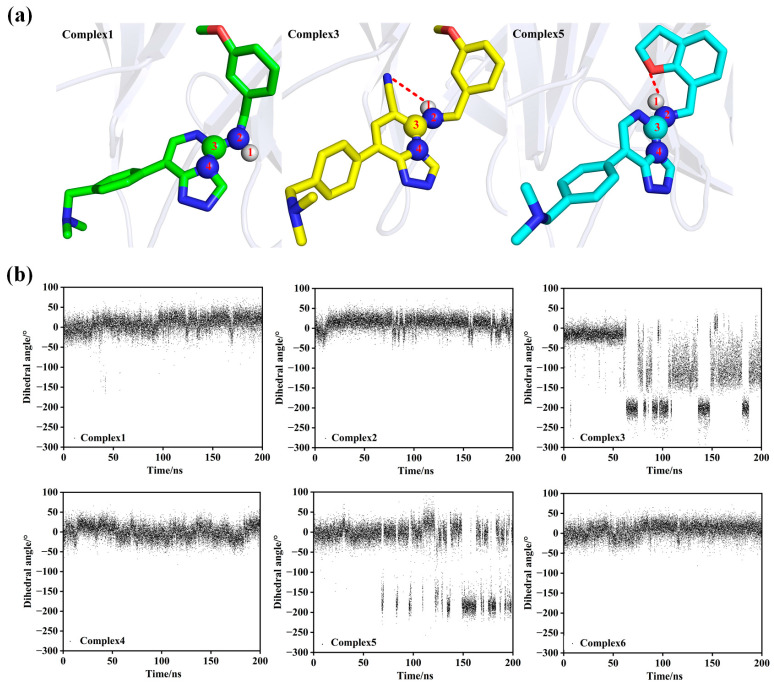
Intramolecular interactions diagrams and dihedral angle diagrams. (**a**) The hydrogen atom on the head imino group of the Inhibitors reversal and intramolecular interactions, where green represents the Inhibitor 1, yellow represents the Inhibitor 3, light blue represents the Inhibition 5 and red dashed lines represent intramolecular interactions. (**b**) The change of dihedral angle H_1_-N_2_-C_3_-N_4_ of the six complex systems with the simulation time during the whole simulation process.

A particular phenomenon noticed when making conformational comparisons was that the orientation of the hydrogen atom on the head imino group differed in **Complexes 3** and **5** than in the other Complexes. Figure 8a shows the 1-hydrogen atoms on the head imino group of **Complexes 3** and **5** are on the opposite side of the C_3_-N_2_ bond with the 4-nitrogen atom on the triazole ring, while the other Complexes are on the same side. To ensure this flip was not an isolated phenomenon in the sample, we counted the H_1_-N_2_-C_3_-N_4_ dihedral angles throughout the simulation. Figure 8b shows that the dihedral angle in the other four Complexes is stable at around 0 degrees. In contrast, **Complexes 3** and **5** had a certain percentage of flips, incredibly **Complex 3** after 50 ns when almost the entire conformation left the vicinity of 0 degrees, and a part of it had even reached about −200 degrees. This phenomenon was consistent with what we observed in our sampled conformations.

**Figure 9:**
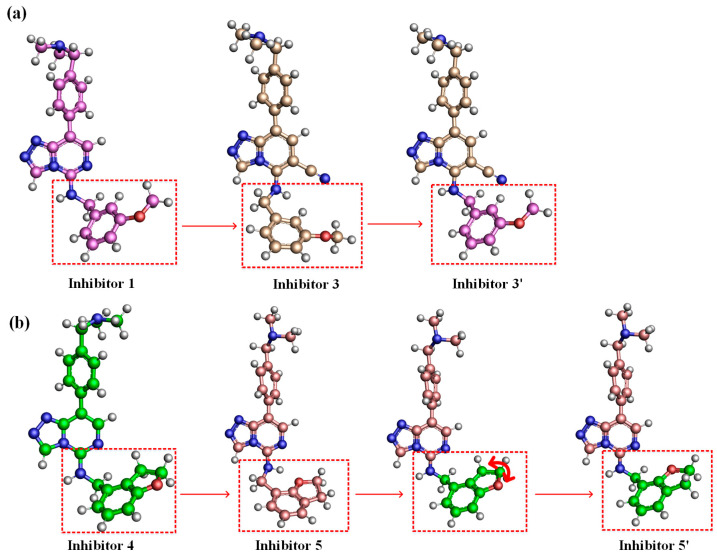
(**a**) Schematic calculation of Gaussian optimization for Inhibitor 3–Inhibitor 3′. (**b**) Schematic calculation of Gaussian optimization for Inhibitor 5–Inhibitor 5′.

The inversion of the hydrogen atom on the head imino group of Inhibitor 3 might result from the more negative charge of the unique cyano group at the 6-position of its parent nucleus, which attracted the positively charged hydrogen atom to the cyano group side (Figure 8a). Comparison of energy of the Inhibitor conformations before and after the hydrogen atom on the head imino group flip using Gaussian 09 calculations could give a quantitative indication of the strength of this intramolecular interaction. Because the head of Inhibitor 1 was the same as that of Inhibitor 3 and the hydrogen atom did not flip, we removed the head of Inhibitor 3. We attached the head of Inhibitor 1 to Inhibitor 3, naming it Inhibitor 3′, as shown in Figure 9a. Then, Gaussian 09 optimized Inhibitors 3 and 3′ at the B3LYP/6-31G(d) level. To prevent the optimized conformations from changing too much, we fixed the atoms with relatively significant conformational changes to keep our calculated conformations the same as those obtained from the simulations. Calculations showed that the energy of Inhibitor 3 was 40.07 kJ·mol^−1^ lower than 3′, indicating this intramolecular interaction made this conformation less energetic when the hydrogen flipped to the cyano group side.

Unlike several other Inhibitors, the negatively charged oxygen atom on the head of Inhibitor 5 was closer to the positively charged hydrogen atom on the head imino group, thus attracting the hydrogen atom to turn to the side nearer to it and forming intramolecular interactions (Figure 8a). Inhibitors 4 and 5 had the same number of atoms but different oxygen atom positions. We removed the head of Inhibitor 5, attached the head of Inhibitor 4 to Inhibitor 5, and exchanged the oxygen atom to the same side as Inhibitor 5, naming it Inhibitor 5′, as shown in Figure 9b. Then, Gaussian 09 optimized Inhibitors 5 and 5′ at the B3LYP/6-31G(d) level. Calculations showed that the energy of Inhibitor 5 was 25.22 kJ·mol^−1^ lower than that of 5′, indicating this intramolecular interaction made the conformation less energetic when the hydrogen atom flipped to the oxygen atom side. The higher energy of the conformational transition of Inhibitor 3 compared to 5 also indicated why there was a more significant flipping ratio of the hydrogen atom on the head imino group of Inhibitor 3 than 5.

The inversion of the hydrogen atom on the head imino group could result in a more considerable distance between the parent nucleus and the head benzene ring, and the head of this class of Inhibitors was relatively fixed in its protein-binding position so that the part of the parent nucleus moved toward the outside of the protein. The previously observed vertical movement of the parent nucleus in Inhibitors 3 and 5 was partly related.

### 2.5. Hydrogen Bond Analysis

The hydrogen bonds between the protein and Inhibitor are often an essential component of affinity. In Table 1, we counted the occurrence rates of hydrogen bonding for some vital residues throughout the simulations. In this case, the statistics for the occurrence of hydrogen bonding for important residues are summed over all types of hydrogen bonds formed by that residue with the inhibitor, and there may be more than one hydrogen bond present in some snapshots, which is why the occurrence of hydrogen bonding for Asn194 exceeds 100% in **Complexes 4** and **6**.

As can be shown in Table 1, there is a significant reduction in the hydrogen bonding occurrence of Asn194 in **Complexes 3** and **5**, which may be related to the inversion of the hydrogen atom on the head imino group in **Complexes 3** and **5**. The reduction in hydrogen bonding might be one of the reasons why the inhibition of these two Inhibitors was lower than that of other Inhibitors. The occurrence rate of hydrogen bonding for other vital residues will be discussed with the binding free energy in Section 2.6 below.

### 2.6. Binding Free Energy Analysis

Binding free energy can determine quantitatively the strength of the interaction between Inhibitor and protein. The six Inhibitors studied in this paper are all competitive, and their inhibition abilities are closely related to their affinity with protein. We used the MM/PBSA method [36] to calculate the binding free energy of six Inhibitors and the EED protein. Various binding free energy contributions between the EED protein and Inhibitors of the six complex systems are shown in Table 2, and the order of their total binding free energy contributions ΔG_binding_ is consistent with the sequence of Inhibitor capabilities measured by experiments. In all complex systems, the Van der Waals Force contribution was much more significant than the electrostatic contribution, indicating that the primary binding of protein to Inhibitors relied on Van der Waals forces.

In addition, Table 2 shows that the Van der Waals Force and electrostatic contributions of **Complex 3** and **Complex 5** are significantly smaller than those of the other Complexes. The decrease in their Van der Waals Force contribution might be due to a change in the Inhibitor binding site, which detached its parent nucleus from the aromatic cage. At the same time, the decrease in electrostatic interactions might be due to the inversion of the hydrogen atom on the head imino group, which reduced the occurrence of hydrogen bonding with Asn194 or the electrostatic interactions with other charged residues.

To identify critical residues in the EED protein that interacted with Inhibitors, we also calculated the decomposition contribution of each residue to the total binding free energy in each of the six systems. Table 3 shows the E_MM_ values of residues with more significant contributions.

**Figure 10:**
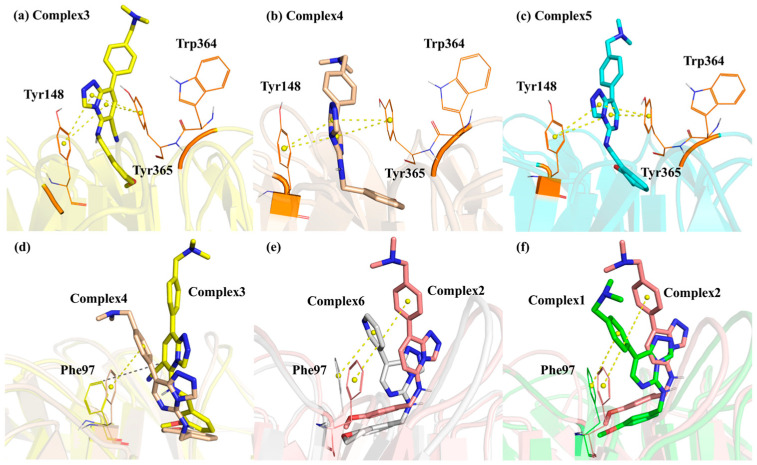
Interaction between aromatic cage residues and Inhibitors, where subfigures (**a**–**c**) are residues Tyr148, Trp364, Tyr365 interacting with the Inhibitor and subfigures (**d**–**f**) are residues Phe97 interacting with the inhibitor. The yellow dashed lines represent π-π stacking interaction, and the grey dashed lines represent hydrophobic interactions.

Table 3 illustrates that the three amino acids Tyr365, Tyr148, and Phe97, which formed the aromatic cage, played a significant role in the E_MM_ of each Complex. These amino acids were the main reason for the inhibitory ability of this class of competitive inhibitors. Conformational observations showed that the aromatic rings of Tyr365 and Tyr148 in all Complexes sandwiched the aromatic ring of the Inhibitor parent nucleus to form a stable sandwich structure (Figure 10a–c). There was a large π-π stacking interaction between these aromatic rings. The E_MM_ contributions of these two residues in **Complexes 3** and **5** (the two weakest Inhibitors) were significantly smaller than in the other four Complexes because the parent nucleus of Inhibitors partially moved away from the aromatic cage, making the π-π stacking interaction between the parent nucleus and Tyr365 and Tyr148 weaker (Figure 10a,c). The E_MM_ contributions of Tyr365 and Tyr148 in Complexes 4 and 6 (the two most potent Inhibitors) were significantly higher than in the other four Complexes, possibly because the extra methylene group on the dihydrofuran ring at the head of the Inhibitors 4 and 6 had more hydrophobic interactions with these two residues than in the other Complexes. Phe97 formed mainly the π-π stacking interaction with the aromatic ring of the Inhibitor tail or hydrophobic interaction with the parent nucleus. Its E_MM_ contributions were also minimized in **Complexes 3** and **5**, as these two Complexes moved outward and disrupted the π-π stacking interaction between Phe97 and the Inhibitor tail (Figure 10d). However, Phe97 had the more considerable E_MM_ contribution in **Complexes 1** and **6**, probably because the benzene rings at the tail in **Complexes 1** and **6** were closer to this residue and formed stronger interactions (Figure 10e,f). The last residue constituting the aromatic cage, Trp364, had a weak E_MM_ contribution in all the Complexes, suggesting that although it was closer to the Inhibitor in position, the spacing of Tyr365 in the middle did not allow it to form more robust interactions with the Inhibitors.

**Figure 11:**
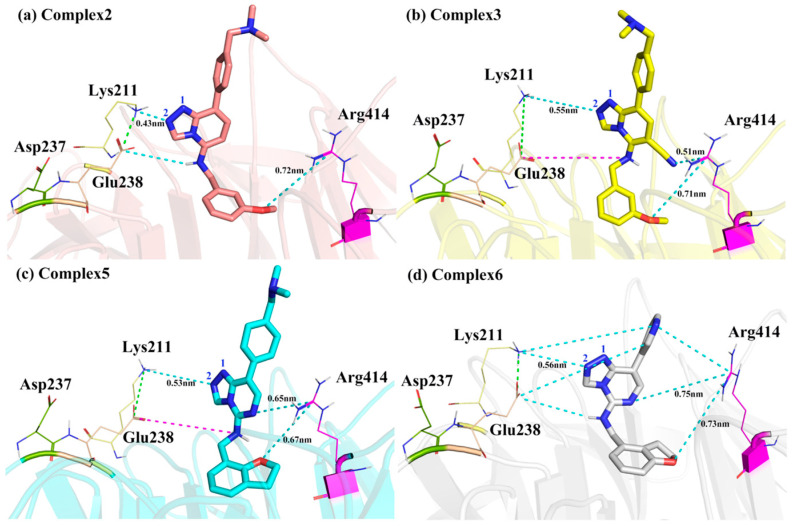
Locations of the four residues interacting with the parent nucleus of the Inhibitors. The green dashed lines represent salt bridges, the blue dashed lines represent the electrostatic attraction, the pink dashed lines represent the electrostatic repulsion and the distances in the figure represent average distances during the simulation.

Lys211, Glu238, and Asp237 were located on the same side of the Inhibitors. The charged side chains of these three residues were very close, where positively charged Lys211 formed a salt bridge with negatively charged Glu238, and Asp237 was next to Glu238, also negatively charged but slightly farther away from the Inhibitors than Glu238 (Figure 11). The group that primarily interacted with Lys211 was the more negatively charged 2-nitrogen atom on the parent nucleus of the Inhibitors (the 1-nitrogen atom also had a weaker interaction). In **Complex 2**, due to the horizontal movement of the Inhibitor parent nucleus in the direction of Lys211, its 2-nitrogen atom on the Inhibitor was closest to the positively charged center nitrogen atom of Lys211 (0.43 nm), and its E_MM_ contribution was the largest (Figure 11a). In **Complexes 4** and **5**, the distance between the 2-nitrogen atom on the Inhibitor and the positively charged center nitrogen atom of Lys211 was not much different (0.50 nm and 0.53 nm), and these two nitrogen atoms could form a certain percentage of hydrogen bonds, whose E_MM_ contribution was close. This distance was relatively far in **Complexes 1**, **3**, and **6** (0.58 nm, 0.55 nm, and 0.56 nm) and could not form hydrogen bonds, so the E_MM_ contributions were similar and smaller.

Glu238 and Asp237, as opposed to Lys211, had negative side charges. The greater the electrostatic attraction exhibited by Lys211, the greater the repulsive force indicated by Glu238 and Asp237, which was more pronounced for Glu238 due to its proximity to the Inhibitor. However, Glu238 in **Complexes 3** and **5** exhibited additional repulsive forces, which might be related to the different orientations of the hydrogen atom on the head imino group. The shift of the hydrogen on the imine with electrostatic attraction to Glu238 to the other side led to a decrease in the electrostatic attraction of the imine with Glu238 and an increase in the repulsive force (Figure 11b,c). In addition, in **Complex 6**, Lys211 and Glu238 exhibited additional electrostatic attraction compared with other Complexes, which might be because the Inhibitor tail was a pyridine ring with more uneven charge distribution, and the charged atoms on the ring could have additional interactions with Lys211 and Glu238 (Figure 11d).

The positively charged guanidine group of Arg414 was located on one side of the pyrimidine ring of the parent nucleus, which was close to the 6-position cyano group on the parent nucleus and the oxygen atom on the head. It could form an electrostatic interaction with the charged groups in these two parts (Figure 11). Among them, the 6-position cyano group in **Complex 3** was the closest to this residue and carried an enormous negative charge on the nitrogen atom of the cyano group (0.53e). Therefore, the E_MM_ contribution of Arg414 in **Complex 3** was the largest (Figure 11b). Because Arg414 was in the outer layer of the EED protein, its more interactions with the 6-position cyano group caused Inhibitor 3 to move outward overall by its attraction, so the vertical movement was more significant in **Complex 3** than in **Complex 5**. In contrast to **Complex 3**, in **Complex 2** with no polar nitrogen atom at the 6-position (the charge on the C-H group was only 0.11e), Arg414 had a minor interaction with the parent nucleus and a little E_MM_ contribution, resulting in Inhibitor 2 being more prone to horizontal movement due to the lack of fixation of this interaction (Figure 11a). The remaining four complexes were all nitrogen atoms at the 6-position, with the E_MM_ contribution of Arg414 intermediate between **Complexes 2** and **3**.

**Figure 12:**
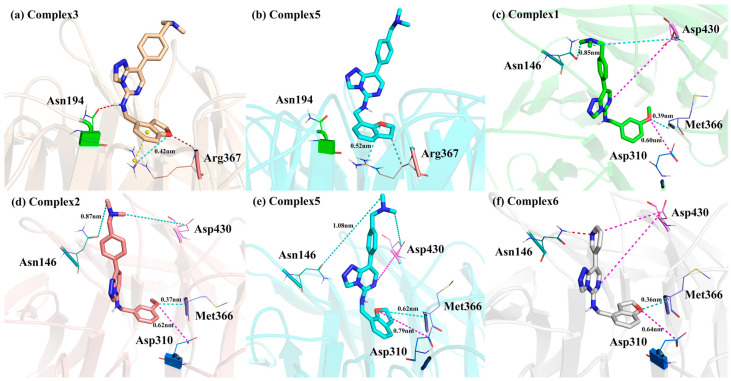
Locations of the six residues interacting with the head and tail of the Inhibitors. The red dashed lines represent the hydrogen bond, the yellow dashed lines represent π-π stacking interaction, the gray dashed lines represent hydrophobic interactions, the blue dashed lines represent the electrostatic attraction, the pink dashed lines represent the electrostatic repulsion and the distances in the figures represent average distances during the simulation.

Asn194 was also the residue with a more considerable E_MM_ contribution to the binding free energy. Previous hydrogen bonding analysis showed that this residue formed more significant occurrence rates of hydrogen bonding with the hydrogen atom on the head imino group of the Inhibitors (Figure 12a). The hydrogen bonding analysis also showed a considerable decrease in the Asn194 hydrogen bonding occurrence rates in **Complexes 3** and **5**, related to the flipping of the hydrogen atom on the head imino group (Figure 12b). Consequently, the E_MM_ contribution of Asn194 was smaller in these two Complexes than in the other four Complexes.

Arg367, Asp310, and Met366 were all located near the heads of the inhibitors, and the first two could form a variety of interactions with charged groups in the inhibitor due to their charged side chains. Among them, the electrostatic interactions were strongest with the oxygen atoms of the head that are near them (Arg367 for electrostatic attraction and Asp310 for repulsive forces). In Met366, on the other hand, it was the -NH- in the peptide plane that formed electrostatic attraction with the head oxygen atom. In **Complex 2**, due to the different positions of the head oxygen atom (as shown in Figure 12), all three residues in this complex exhibit different E_MM_ contributions than in the other complexes.

Asn146 was close to the tails of the Inhibitors. The tail of Inhibitor 6 was not a benzene ring but a pyridine ring (Figure 12d). In the simulation process, the pyridine ring was prone to rotation. When the nitrogen atom with a relatively concentrated negative charge was transferred to the side of Asn146, a certain proportion (17.61%) of a hydrogen bond would be formed with the amino group on Asn146. Therefore, its E_MM_ contribution was more significant. The aromatic rings at the tail of the remaining five Complexes were all benzene rings with relatively dispersed charges. However, they all carried polar trimethylamine groups, which could also interact with Asn146. The tail trimethylamine of Inhibitor 1 was significantly closer to Asn146 than the others (Figure 12c), so its E_MM_ contribution was also more substantial.

Asp430 was located between the parent nucleus 6-position and the tail of the inhibitor. Its negatively charged side chain could exhibit repulsion with the negatively charged group at the 6-position of the parent nucleus. In addition, in **Complex 6**, this residue also exhibited repulsion with the nitrogen atom on the tailed aryl ring of the inhibitor (as shown in Figure 12f), whereas in the other five complexes, it would exhibit electrostatic attraction with the tailed trimethylamine group. Thus, inhibitor 6, which had a nitrogen atom on the tail aromatic ring of the inhibitor and no trimethylamine group, had the greatest repulsive force with Asp430 (as shown by the E_MM_ contribution having the largest positive value). Inhibitor 3, which had the trimethylamine group closest to Asp430, exhibited the greatest electrostatic attraction with Asp430.

## 3. Discussion

### 3.1. Comparison of Inhibitors and Natural Substrate Binding Modes

**Figure 13:**
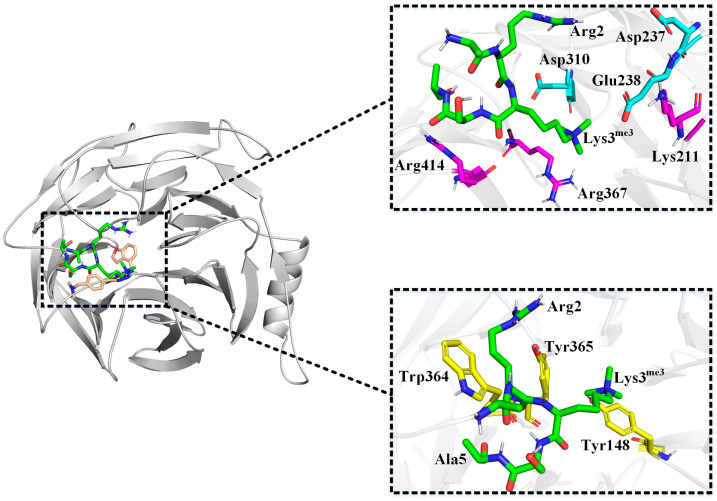
Comparison of ARL^me3^SA and Inhibitor binding poses and interaction of ARL^me3^SA with its surrounding residues. The green portion is ARL^me3^SA and the gold portion is the Inhibitor 4. The light blue residues represent the negatively charged region, the pink residues represent the positively charged region, and the yellow residues represent the aromatic cage.

Comparing the binding modes of inhibitors and natural substrate to EED protein provide more insight into the main sources of inhibitor competitiveness. We performed simulations of EED-ARL^me3^SA and calculated its binding free energy. The results showed that the binding posture of the ARL^me3^SA on EED was quite different from that of the Inhibitors of the triazolopyrimidine derivatives class. This might be due to the large number of residues in the EED protein with charged side chains (44 negatively charged glutamate/aspartate and positively charged lysine/arginine out of a total of 362 residues). Of these, the natural substrate binding was more negatively charged on the top surface and more positively charged on the side of the protein and the bottom of the center pocket. However, the natural substrate had a positive charge on both Arg2 and Lys3^me3^, which repelled the positively charged residues at the bottom of the central pocket, and it did not interact strongly with the aromatic cage due to the relatively small number of nonpolar side chains on the natural substrate. As a result, the Lys^me3^ side chain in the natural substrate was inserted more shallowly into the central pocket compared to the Inhibitors of the triazolopyrimidine derivatives class (as shown in Figure 13) and instead tended to form ionic interactions with the region of negative charge concentration on the top surface.

The results of binding free energy calculation could prove the above conclusions quantitatively. The Van der Waals interaction between the protein and the natural substrate was only −13.95 kJ·mol^−1^, which was much smaller than the electrostatic interaction between the two (−162.93 kJ·mol^−1^), which was the opposite of the results for the six Inhibitors. The decomposition contribution of each residue indicated that the residues that formed the strongest interactions with the natural substrate were those with charged side chains, such as Glu238 (−38.65 kJ·mol^−1^), Asp310 (−30.72 kJ·mol^−1^) and Asp237 (−27.74 kJ·mol^−1^) that exhibited electrostatic attraction, and Lys211 (33.57 kJ·mol^−1^), Arg367 (29.35 kJ·mol^−1^) and Arg414 (28.89 kJ·mol^−1^) that exhibited repulsive forces. The first three of these residues formed a negatively charged pocket on the top surface and were the primary region for binding to the positively charged Arg2 and Lys3^me3^ in the natural substrate. Arg367 was located at the bottom of the central pocket and was the primary residue that resisted the penetration of Lys3^me3^ into the central pocket. In contrast, Tyr365, Trp364, and Tyr148 among the aromatic cage residues formed the largest hydrophobic interactions with the natural substrate, but all of them had smaller E_MM_ contributions of −2.8142 kJ·mol^−1^, −2.2539 kJ·mol^−1^, and −1.6934 kJ·mol^−1^, respectively, with Tyr365 and Tyr148 forming hydrophobic interactions mainly with Lys3^me3^ in the natural substrate and Trp364 mainly formed hydrophobic interactions with the hydrophobic portion on the Arg2 side chain of the natural substrate as well as the side chain methyl group of Ala5 (as shown in Figure 13).

Although ARL^me3^SA formed ionic interactions with proteins with a strength greater than or equal to that of hydrogen bonding and hydrophobic interactions, the simultaneous presence of many positively charged residues in EED protein caused their repulsive forces with ARL^me3^SA to offset a large portion of the electrostatic attraction. Therefore, the ΔG_binding_ of ARL^me3^SA with EED was only −139.14 kJ/mol, which was only lower than the weaker Inhibitors 2 and 5 among the six inhibitors, which provided an opportunity for the development of inhibitors targeting EED.

### 3.2. Differences in the Inhibitory Capacity of the Six Inhibitors

The six Inhibitors studied in our study could be grouped and compared to determine the effect of different parts of Inhibitors on inhibitory ability.

(i) The head and tail of Inhibitors 1,2 and 3 were identical, only the substituents on the 6-position of the parent nucleus were different, and their inhibitory capacities were more than 100 times different. Among them, Inhibitor 3 had the weakest inhibitory ability (IC_50_ = 13,000 nM), and the difference between Inhibitors 1 and 2 was only three times (IC_50_ = 110 nM and 320 nM). In the previous conformation comparison, in Inhibitor 3, the hydrogen atom on the head imino group was prone to flip due to the stronger electrostatic attraction of the cyano group on the 6-position of the parent nucleus. This flip resulted in a decrease in the hydrogen bond formed between it and Asn194, resulting in a considerable weakening of the binding of Inhibitor 3 to Asn194. In addition, the parent nucleus of Inhibitor 3 underwent a maximal vertical movement, which partially removed the parent nucleus and the trailing benzene ring from the optimal binding position with the aromatic cage residues (Phe97, Tyr148, and Tyr365), resulting in a weakening of the binding of the Inhibitor 3 to the aromatic cage residues. There was a significant reduction in the E_MM_ contribution of Phe97. The above results indicate that in the design of this kind of drug, it is necessary to avoid adding strong negatively charged groups to the 6-position of the parent nucleus to prevent the reversal of the hydrogen atom on the head imino group.

The conformation comparison also showed that the parent nucleus of Inhibitor 2 moved horizontally compared to Inhibitor 1 due to the lack of fixation by Arg414. Unlike vertical movement, this horizontal movement did not cause the Inhibitor parent nucleus to leave the aromatic cage, so the binding of Inhibitor 2 to the aromatic cage residues was somewhat reduced but not as significant as in Inhibitor 3. At the same time, the horizontal movement resulted in a significant decrease in the distance between the 2-nitrogen atom on the parent nucleus triazole ring and the charged residues such as Lys211, Asp237, and Glu238, and the overall manifestation was an increase in the binding of Inhibitor 2 to the EED protein. The above interactions cancel each other, and the combined result made the binding free energy of Inhibitor 2 and the EED protein slightly smaller than that of Inhibitor 1, and the inhibition ability is slightly decreased.

(ii) The parent nucleus and tail of Inhibitors 1,4 and 5 were identical; only the head was different. Inhibitors 4 (IC_50_ = 20 nM) and 5 (IC_50_ = 2600 nM) formed a dihydrofuran ring on their head, but the location of the oxygen atom in the ring was different, making their inhibition ability different by 130 times. Previous results had shown that the hydrogen atom on the head imino in Inhibitor 5 was susceptible to flipping due to the intramolecular electrostatic attraction of the head oxygen atom. Like the previous Inhibitor 3, the flipping of the hydrogen atom resulted in a weakening of the binding of Inhibitor 5 to Asn194, as well as a vertical movement of the parent nucleus and tail of Inhibitor 5, and finally a decrease in the binding of Inhibitor 5 to the aromatic cage residues Tyr148 and Phe97. In addition, the difference in the position of the oxygen atom in the head also resulted in a reduced electrostatic attraction between Inhibitor 5 and Arg367 and Met366, which also reduced the affinity between the EED protein and Inhibitor 5.

Inhibitor 1 (IC_50_ = 110 nM) and Inhibitor 4 (IC_50_ = 20 nM) had essentially the same head oxygen atom position but had an open-loop structure in Inhibitor 1. A comparison of the conformations revealed that the head and parent nucleus positions of Inhibitors 1 and 4 overlap almost precisely, but there was a significant difference in the tails. This difference might be due to intramolecular interactions. That is, the position of the tailed benzene ring in Inhibitor 4 was closer to the dihydrofuran ring in the head, forming specific intramolecular hydrophobic interactions. To quantify the stability of the two tail orientations, we attached the benzodihydrofuran ring on the head of Inhibitor 3 to the head of Inhibitor 1. However, the tail of Inhibitor 1 remained unchanged, naming it Inhibitor 1′. We immobilized atoms with significant conformational changes and optimized Inhibitors 1′ and 3 on B3LYP/6-31G(d) using Gaussian 09. The results showed that the energy of Inhibitor 3 was 13.50 kJ·mol^−1^ lower than Inhibitor 1′, suggesting that intramolecular interactions made the conformation of Inhibitor 3 more stable.

The change in the tail conformation of the Inhibitor allowed the tail benzene ring to be fixed in a position that facilitated stable hydrophobic interaction with Tyr365, Trp364, and Tyr148 of the EED protein. As a result, each of these residues bonded more strongly in Inhibitor 3 than in Inhibitor 1. The above results suggest that in drug design, such Inhibitors should not carry a strong negatively charged group at the 8′-position of the head but preferably with a hydrophobic group to maintain a more favorable tail conformation.

(iii) The heads and parent nuclei of Inhibitors 4 (IC_50_ = 20 nM) and 6 (IC_50_ = 24 nM) were identical, but their tails differed. The difference in the binding positions of Inhibitor 4 and Inhibitor 6 in the EED protein was negligible. The E_MM_ contributions showed that the binding of the two Inhibitors with the same parent nucleus to the aromatic cage residues differed little. The different Inhibitor tails mainly affected residues Phe97, Asn146, and Asp430, which were closer to it, as well as residues Arg414, Lys211, Glu238, and Asp237, which were slightly further away but with a charged side chain that could interact with the tail in a long-range electrostatic force. However, these residues positively and negatively affected the binding free energy between the EED and Inhibitor, essentially canceling each other. As a result, the difference in the total binding free energy of the two Complexes was slight, and the experimental results also showed a small difference in the inhibitory capacity of the two Inhibitors.

## 4. Materials and Methods

### 4.1. Acquisition of the EED Protein and Inhibitor Structures

The X-ray crystal diffraction structure (PDBID:7QK4, resolution: 1.6 A) [30] of the EED-MAK683 Complex was obtained from the RCSB database, and the solvent molecules and the original inhibitor MAK683 in the crystal were removed to get the EED protein. Complete the missing residues in the middle of the EED protein using the model loops panel in Chimera [37]. All structures of Inhibitors were constructed from Gaussview 6.0 and optimized at B3LYP/6-31G(d) level using The Gaussian 09 software package to obtain stable initial structure and Mulliken charge [38].

### 4.2. Molecular Docking

We used AutoDock Vina [39,40,41] as molecular docking software to obtain EED protein Complexes with the six Inhibitors we studied. Since these are all competitive Inhibitors [30], we selected the binding pocket of the natural substrate as the docking area, the aromatic cage area mentioned in the introduction. We used the built-in grid of AutoDock Vina to generate a grid box with 24 × 24 × 24 grid points in the x, y, and z directions with a grid spacing of 0.1 nm, and the center coordinates of the grid in the x, y, and z directions were 8.291, 17.934, and 17.401. Then, each of the six Inhibitors was molecular docked with the EED protein in the grid. AutoDock Vina outputted the top 20 site poses for each Inhibitor and selected the conformations that scored high and matched the binding modes reported in the literature as the initial structures for the next step of molecular dynamics simulations. The Complexes obtained from the six docking groups were named Complexes 1–6.

### 4.3. Molecular Dynamics Simulation

Taking the structures of the Complexes obtained by molecular docking as the initial conformations, we performed molecular dynamics simulations of all the complex systems using the Gromacs2018.8 program [42,43,44] As a reference, the same simulation was performed for the EED-ARL^me3^SA complex with natural substrates (PDBID:3IIW). The EED Protein selected the AMBER14SB-parmbsc1.ff force field [45]. The antechamber program in the AMBER16 [46] software package generated the GAFF force field for the Inhibitors [47]. Then, the TIP3P water molecule model [48] was used to solvate the entire complex systems. The shortest distance between the protein edge and the cubic solvent box boundary was 1 nm. The total number of water molecules added after the solvation of different complex systems was about 22,600. To maintain the electrical neutrality of the system, we added two sodium ions to neutralize the negative charge in the protein. The steepest descent method minimized the system’s energy by 1000 steps to ensure no atomic collisions or unreasonable spatial structure. Then the balance of two stages was carried out. The first stage was the NVT balance of 500 ps at 300 K temperature. In the second stage, the NPT balance of 500 ps was performed at the same temperature, and the pressure was set at 1atm. The final product simulations were carried out separately for 200 ns. The integration step was set to 2 fs, and the long-range electrostatic cut-off radius was 1.2 nm. The coordinates and energy information were saved every 10 ps during the simulations. Two sets of parallel simulations were performed for each system to rule out randomization of results.

### 4.4. Binding Free Energy Calculation

We used the Molecular Mechanics/Poisson–Boltzmann surface area (MM/PBSA) method to calculate the binding free energy based on the simulation results [36,49]. A point was selected every 20 frames from the equilibrium trajectory simulated by molecular dynamics, and 1000 frames of conformation were chosen with a duration of 20 ns. The binding free energy of each protein–ligand complex system was calculated using the g_mmpbsa tool in the Gromacs2018.8 software package, and the contribution of each residue to the decomposition of free energy was also estimated. The binding free energy of protein-ligand complexes is defined as follows [50]:

ΔG_bind_ = ⟨G_complex_⟩ − ⟨G_protein_⟩ − ⟨G_ligand_⟩ (1)

G_complex_, G_protein,_ and G_ligand_ represent the free energy of the protein–ligand complex, protein, and ligand. The individual entity free energies of each of them can be further described as the following:

G_x_ = ⟨E_MM_⟩ − TS + ⟨G_solv_⟩ (2)

where x stands for complex, protein, and ligand, the free energy G is divided into two terms, namely molecular mechanical term and solvation energy. <E_MM_> represents the potential energy of molecules in a vacuum, <G_solv_> represents the free energy of solvation, and TS refers to the contribution of entropy to the free energy in a vacuum. In the Gromacs program, the TS term is approximately zero.

The vacuum molecular mechanical potential energy (E_MM_) is the sum of the energy of E_bonded_ and E_nonbonded_ interactions, as shown in the following equation:

E_MM_ = E_bonded_ + E_nonbonded_ (3)

E_bonded_ and E_nonbonded_ can also be expressed as:

E_bonded_ = E_bond_ + E_angle_ + E_torsion_ (4)

E_nonbonded_ = E_elec_ + E_vdW_ (5)

where E_bond_, E_angle_, and E_torsion_ are represented as bonding interactions of bond length, bond angle, and dihedral angle, while E_elec_ and E_vdW_ are represented as non-bonding interactions of the Van der Waals Force and the Coulomb Force and are modeled using the Coulomb Force potential function and Lennard–Jones potential function respectively.

The free energy of solvation (G_solv_) is composed of the polar free energy of solvation (G_polar_) and non-polar free energy of solvation (G_nonpolar_), which can be expressed as the following formula:

G_solv_ = G_polar_ + G_nonpolar_ (6)

## 5. Conclusions

In this work, molecular dynamics simulations and binding free energy calculations were performed on six triazolopyrimidine inhibitors and natural substrates in complexes with the EED subunit in PRC2. We mainly concluded the following: on the one hand, we compared the inhibitor to a positively charged natural substrate due to the high number of positively charged residues in the central pocket of the EED, making the binding of the natural substrate to the EED unstable and providing an energetic basis for competition for the inhibitor. On the other hand, we found that when the 6-position of the parent nucleus or the 8′-position of the head benzodihydrofuran had a strong negatively charged group, it caused the hydrogen atom on the head imino group to be susceptible to flipping and the parent nucleus moving vertically, resulting in a significant decrease in inhibitory ability. Furthermore, when the 6-position of the parent nucleus was a nonpolar group, its reduced electrostatic interaction with Arg414 resulted in the parent nucleus moving horizontally, which decreased the inhibitory ability slightly. We also discovered that when the head formed a benzodihydrofuran ring and the 8′-position was methylene, the inhibitory ability was significantly improved. Finally, we found that the differences in the tail of the inhibitor had little impact on its inhibitory ability.

## Figures and Tables

**Table 1:** Occurrence of vital residues in protein forming hydrogen bonds with Inhibitors.

Complex	Bonding Residue	Occurrence (%)
Complex 1	Asn194	85.34
	ARG367	8.47
	ASN146	1.33
Complex 2	ASN194	98.24
	ARG367	8.34
	LYS211	6.06
Complex 3	ASN194	37.28
	ARG414	19.18
	ARG367	4.14
Complex 4	ASN194	108.51
	ARG367	6.65
	LYS211	3.70
Complex 5	ASN194	63.60
	ARG414	4.57
	LYS211	4.10
Complex 6	ASN194	104.13
	ASN146	17.61
	ARG367	6.12

**Table 2:** Binding free energy of six compounds calculated by MM/PBSA (kJ·mol^−1^).

Energy Contribution	Complex 1	Complex 2	Complex 3	Complex 4	Complex 5	Complex 6
ΔE_vdW_	−209.73	−192.71	−150.48	−227.44	−176.55	−219.95
ΔE_elec_	−76.93	−79.11	−45.68	−83.26	−37.68	−93.09
ΔG_PB_	151.89	145.65	100.42	154.29	111.65	159.39
ΔG_SA_	−17.66	−16.22	−12.07	−16.87	−13.74	−17.34
ΔG_polar_^a^	74.96	66.54	54.74	71.03	73.97	66.30
ΔG_nonpolar_^b^	−227.39	−208.93	−162.55	−244.31	−190.29	−237.29
ΔG_binding_^c^	−152.43	−142.39	−107.81	−173.28	−116.32	−170.99

^a^ ΔG_polar_ = ΔG_elec_ + ΔG_PB_, ^b^ ΔG_nonpolar_ = ΔG_vdW_ + ΔG_SA_, ^c^ ΔG_bind_ = ΔG_polar_ + ΔG_nonpolar_.

**Table 3:** E_MM_ contributions of significant residues (kJ·mol^−1^).

Residue	Complex 1	Complex 2	Complex 3	Complex 4	Complex 5	Complex 6
Tyr365	−22.04	−25.58	−21.86	−27.71	−22.18	−27.76
Tyr148	−16.80	−14.11	−13.19	−17.36	−12.77	−18.74
Phe97	−16.03	−12.24	−8.53	−14.50	−10.94	−17.06
Trp364	−1.38	−2.21	−1.26	−3.67	−2.02	−1.74
Arg367	−8.80	−4.83	−7.61	−9.99	−0.99	−7.65
Asn194	−20.71	−20.27	−6.99	−22.94	−11.15	−23.50
Lys211	−5.56	−21.37	−5.24	−11.64	−12.93	−5.90
Glu238	−3.29	4.28	−0.84	−0.92	5.41	−5.84
Asp237	0.53	2.65	0.10	1.02	1.37	−0.20
Arg414	−3.53	−1.91	−6.86	−2.80	−5.63	−5.02
Asp310	−3.54	−4.21	−3.34	−3.34	−10.85	−4.37
Met366	−3.61	−4.02	−2.35	−5.83	−1.05	−5.59
Asn146	−4.68	−2.39	−2.84	−1.71	−1.55	−4.15
Asp430	−1.25	−1.68	0.20	−3.95	−0.89	1.50

## Data Availability

Data are contained within the article and Supplementary Materials.

## Supplementary Materials

The following supporting information can be downloaded at: https://www.mdpi.com/article/10.3390/molecules28247997/s1.
